# More similarity than difference: Comparison of within- and between-sex variance in early adolescent brain structure

**DOI:** 10.1162/IMAG.a.127

**Published:** 2025-09-02

**Authors:** Carinna Torgerson, Katherine Bottenhorn, Hedyeh Ahmadi, Jeiran Choupan, Megan M. Herting

**Affiliations:** Department of Population and Public Health Sciences, University of Southern California, Los Angeles, CA, United States; Mark and Mary Stevens Neuroimaging and Informatics Institute, University of Southern California, Los Angeles, CA, United States; NeuroScope Inc., New York, NY, United States

**Keywords:** neuroimaging, sex differences, white matter, gray matter, diffusion, adolescence

## Abstract

Adolescent neuroimaging studies of sex differences in the human brain predominantly examine average differences between males and females. This focus on mean differences without probing relative distributions and similarities may contribute to both conflation and overestimation of sex differences and sexual dimorphism in the developing human brain. We aimed to characterize the variance in brain macro- and micro-structure in early adolescence as it pertains to sex at birth using a large sample of 9–11-year-olds from the Adolescent Brain Cognitive Development (ABCD) Study (N = 7,723). For global and regional estimates of gray and white matter volume, cortical thickness, and white matter microstructure (i.e., fractional anisotropy and mean diffusivity), we examined: within- and between-sex variance, overlap between male and female distributions, inhomogeneity of variance, effect size, and CLES. We examined these sex differences using both unadjusted (raw) brain estimates and residualized brain estimates from mixed-effects modeling (adjusted) to account for variance better attributed to age, pubertal development, socioeconomic status, race, ethnicity, MRI scanner manufacturer, and total brain volume, where applicable. Contrary to the popular view of the brain as sexually dimorphic, we found high similarity and low difference between sexes in all regional measurements of brain structure examined after accounting for other sources of variance. However, the sex difference for adjusted total brain volume (TBV) had a medium effect size and a 71.9% probability that a randomly chosen male adolescent would have a larger brain than a randomly chosen female adolescent. All cortical and subcortical volumes showed significant inhomogeneity of variance between sexes, whereas a minority of brain regions showed significant sex differences in variance for cortical thickness, white matter volume, fractional anisotropy, and mean diffusivity. Previously reported sex differences in early adolescent regional human brain volume may, therefore, be driven by disparities in variance, rather than binary, sex-based phenotypes. This study builds upon previous findings illustrating the importance of considering variance when examining sex differences in brain structure.

## 1 Introduction

Sexual dimorphism refers to traits with two distinct forms, each existing predominantly or exclusively among one sex, whereas sex differences describe traits that fall along a continuum, but exhibit a difference in mean or variability between males and females (DeCasien et al., 2022; McCarthy et al., 2012). In the neuroscience literature, the conflation of the terms is exacerbated by researchers’ tendency to focus on mean sex differences. For example, when interpreting sex differences, the mean trait or phenotype is often generalized to the entire sex (i.e., “males have larger brains than females”) (Sanchis-Segura et al., 2022). A tenet of this area of research is that sexual differentiation may occur during the fetal period as well as other sensitive windows of brain maturation. As such, early adolescence—a peripubertal period of rapid growth with high inter-individual variance—has become a crucial period of study for the development of sex differences in the brain (Beck et al., 2023; Brennan et al., 2021; Kaczkurkin et al., 2019; Peper et al., 2020).

Adolescent studies of sex differences in brain structure predominantly test for significant mean group differences between males and females (Giedd & Denker, 2015; Giedd et al., 2012; Kaczkurkin et al., 2019; Lenroot & Giedd, 2010). On average, regional cortical volumes are larger among male adolescents than among female adolescents (Gennatas et al., 2017; Paus et al., 2010), as are a number of subcortical regions, including the putamen, pallidum, amygdala, thalamus, and cerebellum (Adeli et al., 2020; Paus, 2010; Paus et al., 2010). However, some authors have reported greater whole-brain cortical thickness in adolescent females than in males (Zhou et al., 2015), while others reported no sex differences (Bramen et al., 2012; Menary et al., 2013; Vijayakumar et al., 2016). In addition to higher gray matter volume, male adolescents also display higher white matter volumes relative to female adolescents (Pfefferbaum et al., 2016). Studies of fractional anisotropy (FA) and mean diffusivity (MD)—measures of white matter microstructure commonly used to study white matter development and integrity—have shown mixed results. For example, some studies report higher FA in male adolescents compared to females (Herting et al., 2012; Lawrence et al., 2023; Pohl et al., 2016; Torgerson et al., 2024) while others report higher FA in female adolescents (Bava et al., 2011; Schmithorst et al., 2007). However, females enter puberty and reach maturity at younger ages than males (Brix et al., 2019). Similarly, measures of gray and white matter structure peak earlier in girls than in boys (Raznahan et al., 2011; Simmonds et al., 2014). Therefore, it is important to account for differences in both maturation and chronological age when studying peripubertal development.

Despite relatively small effect sizes, numerous studies have concluded that these differences amount to sexual dimorphism in the developing brain (Brennan et al., 2021; Herting et al., 2012; Lenroot et al., 2007; Paus et al., 2010; Seunarine et al., 2016; Yang et al., 2021). This elevation of sex differences to sexual dimorphism inappropriately uses aggregate statistical results to infer the nature of inter-individual relationships (Eliot et al., 2021; Joel & Fausto-Sterling, 2016; Maney, 2016; Sanchis-Segura & Wilcox, 2024), which is a form of ecological fallacy (Gnaldi et al., 2018; Nieri et al., 2003; Paik, 1985). For example, though males have—on average—9–10% larger brains in adolescence (Giedd et al., 1997, 2015; Lenroot et al., 2007), this statistic alone does not indicate that a randomly selected female is more likely than not to have a regional brain volume below a randomly selected male or below the population mean. Similarly, a mean sex difference is not sufficient evidence to claim that all females are more similar to each other than to any males. Such a comparison would require a deeper understanding of the dispersion of the data, particularly the relative within- and between-sex variance (Warton & Hui, 2017). Therefore, more nuanced statistical approaches are required to more fully contextualize the sex differences noted in the existing neuroimaging literature. In fact, in adults, overlap distribution statistics and formal analyses of similarity have shown extensive overlap between the distributions of MRI brain outcomes for each sex (N = 1,403; total age ranges 12–75 years) (Joel et al., 2015) and that brain metrics from two random individuals of the same sex differ as much as those from two random individuals of the opposite sex (Sanchis-Segura et al., 2022). These innovative statistical approaches challenge the narrative of “hard-wired” differences between “male brains” and “female brains” (Amen, 2013; Baron-Cohen, 2009; Blum, 1998; Brizendine, 2006, 2022; Darlington, 2009; Gurian, 2010; Gurian & Stevens, 2006; James, 2009; Lundin, 2009; McKay, 2018; Schulz, 2005). However, similar research contextualizing sex differences in child and adolescent brains remains sparse.

Using the largest study of brain development—the Adolescent Brain Cognitive Development℠ Study (ABCD Study®)—we recently examined how sex and gender relate to gray matter macrostructure and white matter microstructure in a nationwide U.S. sample of 9–11-year-olds (Torgerson et al., 2024). We found that sex—but not felt-gender—was a significant predictor of early adolescent subcortical volume, cortical thickness, local gyrification index, and white matter microstructure in the majority of regions examined. Furthermore, Wierenga et al. (2018, 2022) previously found that male variability in the volumes of the hippocampus, pallidum, putamen, and cerebral gray and white matter was greater than female variability not only at the sample mean, but also at the extremes upper and lower ends of the distribution for children and adolescents. Building on this work, we examined inter-individual variability in brain development in the ABCD Study and found sex differences in the variability of the annualized percent change (Bottenhorn et al., 2023). Specifically, we reported greater male variability in white matter volumes and network connectivity, but greater female variability in the development of cortical macro- and micro-structure between the ages of 9 and 13 years (Bottenhorn et al., 2023).

Consequently, this study aims to contextualize the cross-sectional relationship between mean group sex differences and inter-individual differences in brain structure in early adolescents ages 9 to 11 years old. Building upon our previous findings showing widespread, yet very small effect sizes (Torgerson et al., 2024), we aimed to further characterize within- and between-group differences, inhomogeneity between the sexes, overlap between the sexes, and effect size for sex across various macro- and micro-structural brain metrics. Given large differences in overall head sizes and other potential confounders, we conducted our analyses on both raw (unadjusted) brain metrics and after adjusting for total brain volume (TBV) and other sociodemographic factors. Based on previous literature (Sanchis-Segura et al., 2022; Wierenga et al., 2018), we hypothesized that the variance between the male and female means would not exceed the within-sex variance, and that this would be true of more regions after adjusting for covariates. We expected inhomogeneity of variance between males and females, in line with previous research (Bottenhorn et al., 2023; Wierenga et al., 2018). In terms of overlap, we hypothesized that we would find high overlap of male and female distributions in all regions examined, and that this overlap would be larger after adjusting for potential confounders, including TBV for volumetric outcomes.

## 2 Methods

### 2.1 Participants

This study utilized data collected as part of the larger ongoing Adolescent Brain Cognitive Development (ABCD) Study®, which involves 11,880 children at 21 different sites around the United States (ABCD Study, 2022; Casey et al., 2018; Hagler et al., 2019). The study included children from diverse geographic, demographic, and socioeconomic backgrounds (Garavan et al., 2018; Heeringa & Berglund, 2020). Children with severe sensory, neurological, medical, or intellectual limitations, lack of English proficiency, or inability to complete an MRI scan were excluded from the ABCD Study (Li et al., 2021). With respect to age, sex, and household size, the ABCD cohort closely matches the distribution of 9–11-year-olds in the American Community Survey, a large probability sample survey of U.S. households conducted annually by the U.S. Bureau of Census (Heeringa & Berglund, 2020). Raw and minimally processed data are publicly available from the ABCD Study in service of increasing reproducibility. We utilized a combination of raw and tabulated questionnaire and neuroimaging data from the study baseline as obtained from the NDA 3.0 (raw T1 and T2 structural MRI files) and 4.0 (tabulated questionnaire and diffusion MRI) releases (NDA 3.0 and 4.0 data release 2021; https://dx.doi.org/10.15154/1523041). We chose to perform our own preprocessing for gray matter macrostructure using both T1w and T2w images to improve parcellation accuracy (Torgerson et al., 2024).

After obtaining the data, we implemented a series of quality control standards (Supplementary Fig. 1). Participants were excluded if their data were collected outside the 21 primary research sites, failed execution of the pre-processing or processing pipelines, failed to meet the raw or post-processing quality control standards of the ABCD consortium (Hagler et al., 2019), or had incidental neurological findings noted by a radiologist (Li et al., 2021). To reduce within-family correlation and meet statistical assumptions for independence, we decided to restrict our sample to one child per family (chosen randomly).

### 2.2 Sex

The ABCD Study collects parent-reported sex assigned at birth. However, due to the multidimensional nature of sex, assignment at birth is not always an accurate reflection of chromosomal sex. Therefore, we also chose to use the frequency ratio of X and Y alleles to detect the presence of a Y chromosome and ascertain the genetic sex of participants. Children whose assigned sex and genetic sex did not match (n = 9) were excluded from the analysis.

### 2.3 Neuroimaging data

A harmonized data collection protocol was utilized across sites with either a Siemens, Phillips, or GE 3T MRI scanner. Motion compliance training, as well as real-time, prospective motion correction was used to reduce motion distortion (Hagler et al., 2019). T1-weighted images were acquired using a magnetization-prepared rapid acquisition gradient echo (MPRAGE) sequence (TR = 2500, TE = 2.88, flip angle = 8), and T2-weighted images were obtained with fast spin echo sequence (TR = 3200, TE = 565, variable flip angle), with 176 slices with 1 mm^3^ isotropic resolution (Casey et al., 2018). Diffusion MRI data were acquired in the axial plane at 1.7 mm^3^ isotropic resolution with multiband acceleration factor 3. Ninety-six non-collinear gradient directions were collected with seven b0 images. Trained technicians inspected T1w, T2w, and dMRI images using a centralized quality control process in order to identify severe artifacts or irregularities (Hagler et al., 2019).

To assess gray matter macrostructure, we obtained baseline T1w and T2w images from the ABCD 3.0 release (NDA 3.0 data release 2020; https://dx.doi.org/10.15154/1520591) and implemented the Human Connectome Project minimal preprocessing pipeline (Glasser et al., 2013) at the Stevens Institute of Neuroimaging and Informatics. Regional parcellation and segmentation were then performed based on the Desikan-Killiany atlas in FreeSurfer 7.1.1 for each participant using T1w and T2w images (Desikan et al., 2006). The primary outcomes of interest included gray matter volume, thickness, and white matter volume in 68 cortical regions, the volume of 20 subcortical regions, as well as FA and MD for 21 white matter tracts (Hagler Jr. et al., 2009). For a complete list of regions by feature, please see Supplementary Table 1.

Tabulated white matter microstructure and demographic data from the baseline study visit were obtained from the 4.0 data release via the NIMH Data Archive (https://nda.nih.gov/abcd/; http://dx.doi.org/10.15154/1523041). ABCD diffusion processing employs five iterations of eddy current correction and robust tensor fitting to minimize gradient distortions and motion (Hagler et al., 2019; Hagler Jr et al., 2009). The b = 0 images are coarsely registered to a diffusion atlas before being registered to T1w images via mutual information. DMRI images are then resampled and registered using the transform from rigid registration of the T1w image to the diffusion atlas. Finally, the diffusion gradient matrix is adjusted for head rotation. Probabilistic atlas-based tractography is then performed with AtlasTrack using a priori tract location probabilities to inform fiber selection (Hagler Jr et al., 2009). For this study, we utilized the tabulated FA and MD data from the AtlasTrack fiber atlas. Specifically, we selected the fornix, cingulate cingulum, parahippocampal cingulum, uncinate fasciculus, superior longitudinal fasciculus, inferior longitudinal fasciculus, inferior fronto-occipital fasciculus, anterior thalamic radiations, corticospinal tracts, forceps major, forceps minor, and corpus callosum as regions of interest (ROIs).

### 2.4 Analyses

All statistical analyses were conducted in R (R Core Team, 2019) with the vegan (Oksanen et al., 2022), lme4 (Bates et al., 2015), effectsize (Ben-Shachar et al., 2020), effsize (Torchiano, 2020), and bayestestR (Markowski et al., 2019) packages. For each ROI, we first calculated some descriptive statistics to characterize the complete distributions of males and females for each measure of brain structure. We began by comparing the within-sex variance to the variance between group means to determine whether the differences between sexes exceeded the differences within each sex for each ROI. Next, we used a robust coefficient of variation (RCV_M_) to compare the relative dispersion of male and female adolescents. We chose to use RCV_M_ as compared to other metrics of coefficient of variation as it is appropriate for use with non-normal data and is robust against outliers (Arachchige et al., 2020). The RCV_M_ was calculated by taking the mean absolute deviation divided by the median and multiplied by a scaling factor of 1.4826 to preserve interpretability relative to the standard coefficient of variation. To contrast with more traditional approaches that focus only on differences, we then examined the similarity between sexes by calculating the overlap coefficient (OVL_2_) using the bayestestR package in R (Markowski et al., 2019), which integrates the minimum density across both distributions and uses the combined x-axis range to measure the percentage of the sample that falls within the overlap between two distributions (Del Giudice, 2022). The bayestestR package’s use of kernel density estimation allows for flexibility in the underlying data distribution to avoid strict assumptions of normality.

To quantify and statistically test the patterns observed in our descriptive statistics, we then compared the male and female distributions using analytical statistics for each global and regional ROI. We chose to investigate inhomogeneity of variance between the sexes with the Fligner-Killeen test, which compares the variances of two groups using a median-centered chi-square (χ^2^) test (Fligner & Killeen, 1976). In order to examine the magnitude of the effect for sex across the entire distribution (rather than just at the group means), we computed Cliff’s delta (δ) statistic using the effsize package (Torchiano, 2020), which estimates the probability that a randomly selected observation from one group is larger than a randomly selected observation from another group, minus the reverse probability (Cliff, 2014; Sanchis-Segura & Wilcox, 2024; Wilcox, 2022). The Cliff’s δ statistic is, therefore, used as a robust, non-parametric measure of the between-sex effect size. The magnitude of Cliff’s δ was interpreted using the thresholds provided by Romano et al. (2006) with δ > 0.474 reflecting a large effect, δ > 0.33 as a medium effect, δ > 0.147 as a small effect, and a δ < 0.147 as negligible.

To aid in the interpretability of these analytical results, we then calculated the common language effect size (CLES)—the probability that a randomly chosen participant would have an observed value greater than a randomly chosen participant of the opposite sex. The computation of Cliff’s δ involves creating a dominance matrix of pairwise comparisons between all males and females. From this dominance matrix, we calculated the probability that a random male adolescent would have a higher value than a random female adolescent and vice versa, as well as the probability that a random male and female would have exactly equal values. Since the probability of equal male and female measures being exactly equal was <0.5% in all regions (max CLES = 0.003), the CLES for one sex can be easily deduced by subtracting the CLES of the other sex from 100; therefore, since the male mean was greater than the female mean in the majority of regional measures, we only report the probability that a randomly selected male subject will have a value larger than a randomly selected female for our CLES results.

To ascertain whether the observed sex differences were partially driven by variance in other variables, we repeated these analyses using residuals of brain outcomes after adjusting for additional independent variables as fixed effects along with data collection site as a random effect (to adjust for the nesting of subjects within sites) (Supplementary Table 2).

Residuals for each brain outcome were obtained from linear mixed modeling using the lme4 package in R (Bates et al., 2015; R Core Team, 2019). To account for additional sources of neuroanatomical variance beyond sex alone as well as site effects, the models included age, pubertal development, race, ethnicity, parent education, scanner, site, and, where appropriate, TBV (see details below). Age was measured in months and rounded to the nearest whole month. Pubertal development was assessed using the parent-report version of the Pubertal Development Scale (PDS) and categorized as prepuberty, early puberty, mid-puberty, late puberty, and post-puberty (Cheng et al., 2021; Herting et al., 2020; Petersen et al., 1988; Thijssen et al., 2020). Since few children in this age range were in late puberty or post-pubertal, we combined the mid-, late, and post-puberty groups into a single category (mid/late puberty). We chose to include measures of race, ethnicity, and socioeconomic status in our models because human neurodevelopment is sensitive to various ecological factors which, due to systemic social injustice, are correlated with sociocultural variables, such as race, ethnicity, and socioeconomic status (Nketia et al., 2021; Werchan & Amso, 2017). Youth race was collected via caregiver report, and caregivers were encouraged to select all answers that applied. Where more than one race was selected, we categorized participants as multiracial Black (if one of their selections was “Black”) or multiracial non-Black. Due to low group numbers, we combined Asian Indian, Chinese, Filipino/a, Japanese, Korean, Vietnamese, Other Asian, American Indian/Native American, Alaska Native, Native Hawaiian, Guamanian, Samoan, other Pacific Islander, and “other race” into a single category (“other race”). Youth ethnicity was parent-reported as either Hispanic or non-Hispanic. To encapsulate socio-economic status, we included educational attainment, operationalized as the highest level of education achieved in the household, and binned into the following categories: less than high school diploma, high school diploma or GED, some college, bachelor’s degree, or postgraduate degree. Idiosyncrasies of different MRI software and hardware can also impact brain segmentation (Liu et al., 2020), so we also included scanner manufacturer (Philips, Siemens, or GE) as a covariate.

Lastly, we conducted several sensitivity analyses to evaluate the influence of TBV and pubertal development on our modeling results. Although it is a common practice to account for the relationship between regional volumes and whole-brain volume (Sanchis-Segura et al., 2020), studies of white matter microstructure generally do not adjust for whole-brain volume (Lebel et al., 2019; Takao et al., 2011). However, our recent findings in the ABCD cohort suggest adjusting for TBV can influence reported sex differences in FA and MD as well (Torgerson et al., 2024). Therefore, we also elected to include and report adjusted models including TBV as a fixed effect as our primary approach, but conducted an additional set of white matter sensitivity analyses using the residuals without TBV in the models. TBV was calculated by FreeSurfer, then scaled by the sample root-mean-square. Since pubertal development differed significantly between boys and girls in our study sample, we re-ran the original models (e.g. including TBV) for all global and regional measures without pubertal development. The results from these sensitivity analyses are reported separately and included as Supplementary findings in Supplementary Tables 3, 5, 7, 9, 11, 13 and 15.

## 3 Results

A full description of the final sample for the current study can be found in Table 1. After stringent data cleaning, our final sample closely matched the full ABCD Study sample in terms of sex, age, pubertal development, and ethnicity, but differed significantly in terms of race and parental education. A demographic comparison of male and female participants in our sample is provided in Table 2. A Chi-square test for independence found differences in pubertal development between male and female participants, with a greater proportion of female adolescents in mid-to-late puberty. Although a *t*-test showed a significant difference in age between male and female participants, the mean and median age was the same for both groups.

**Table 1.** Demographic comparison between all ABCD Study subjects and the study sample.

	ABCD cohort (N = 11,876)	Study sample (N = 7,723)
Sex		
Female	5,680 (47.8%)	3,714 (48.1%)
Male	6,196 (52.2%)	4,009 (51.9%)
Age (months)		
Mean (SD)	118.98 (7.50)	118.99 (7.44)
Median [min, max]	119 [107, 133]	119 [107, 133]
Pubertal development		
Pre	5,837 (49.1%)	3,938 (51.0%)
Early	2,713 (22.8%)	1,860 (24.1%)
Mid/late	2,854 (24.0%)	1,925 (24.9%)
Missing	472 (4.0%)	0 (0%)
Race*		
White	7,517 (63.3%)	5,146 (66.6%)
Black	1,868 (15.7%)	1,062 (13.8%)
Multiracial (Black)	649 (5.5%)	408 (5.3%)
Multiracial (non-Black)	785 (6.6%)	516 (6.7%)
Other^a^	874 (7.4%)	591 (7.7%)
Missing	183 (1.5%)	0 (0%)
Ethnicity		
Non-Hispanic	9,312 (78.4%)	6,147 (79.6%)
Hispanic	2,411 (20.3%)	1,576 (20.4%)
Missing	153 (1.3%)	0 (0%)
Parent education*		
<High school diploma	578 (4.9%)	296 (3.8%)
HS diploma or GED	1,110 (9.3%)	603 (7.8%)
Some college	3,058 (25.7%)	1,970 (25.5%)
Bachelor	3,010 (25.3%)	2,021 (26.2%)
Post graduate degree	4,041 (34.0%)	2,833 (36.7%)
Missing	79 (0.7%)	0 (0%)

* Difference between the research sample and the ABCD Study sample is statistically significant at the p < 0.05 level.

a The “Other” race/ethnicity category includes participants who were parent-identified as Asian Indian, Chinese, Filipino/a, Japanese, Korean, Vietnamese, Other Asian, American Indian/Native American, Alaska Native, Native Hawaiian, Guamanian, Samoan, Other Pacific Islander, or Other Race.

**Table 2.** Demographic comparison between female and male adolescents in the study sample.

	Female (N = 3,714)	Male (N = 4,009)	P-value
Age (months)*			0.027
Mean (SD)	118.80 (7.39)	119.17 (7.47)	
Median [min, max]	119 [107, 132]	119 [107, 133]	
Pubertal development *			**<0.001**
Pre	1,125 (30.3%)	2,813 (70.2%)	
Early	890 (24.0%)	970 (24.2%)	
Mid/late	1,699 (45.7%)	226 (5.6%)	
Race			0.191
White	2,426 (65.3%)	2,720 (67.8%)	
Black	541 (14.6%)	521 (13.0%)	
Multiracial (Black)	202 (5.4%)	206 (5.1%)	
Multiracial (non-Black)	256 (6.9%)	260 (6.5%)	
Other^a^	289 (7.8%)	302 (7.5%)	
Ethnicity			0.787
Non-Hispanic	2,961 (79.7%)	3,186 (79.5%)	
Hispanic	753 (20.3%)	823 (20.5%)	
Parent education			0.395
<High school diploma	158 (4.3%)	138 (3.4%)	
HS diploma or GED	290 (7.8%)	313 (7.8%)	
Some college	934 (25.1%)	1,036 (25.8%)	
Bachelor	960 (25.8%)	1,061 (26.5%)	
Post graduate degree	1,372 (36.9%)	1,461 (36.4%)	

* Difference between the research sample and the ABCD Study sample is statistically significant at the p < 0.05 level.

a The “Other” race/ethnicity category includes participants who were parent-identified as Asian Indian, Chinese, Filipino/a, Japanese, Korean, Vietnamese, Other Asian, American Indian/Native American, Alaska Native, Native Hawaiian, Guamanian, Samoan, Other Pacific Islander, or Other Race.

### 3.1 Global brain measures

#### 3.1.1 Descriptive statistics

**Fig. 1.**
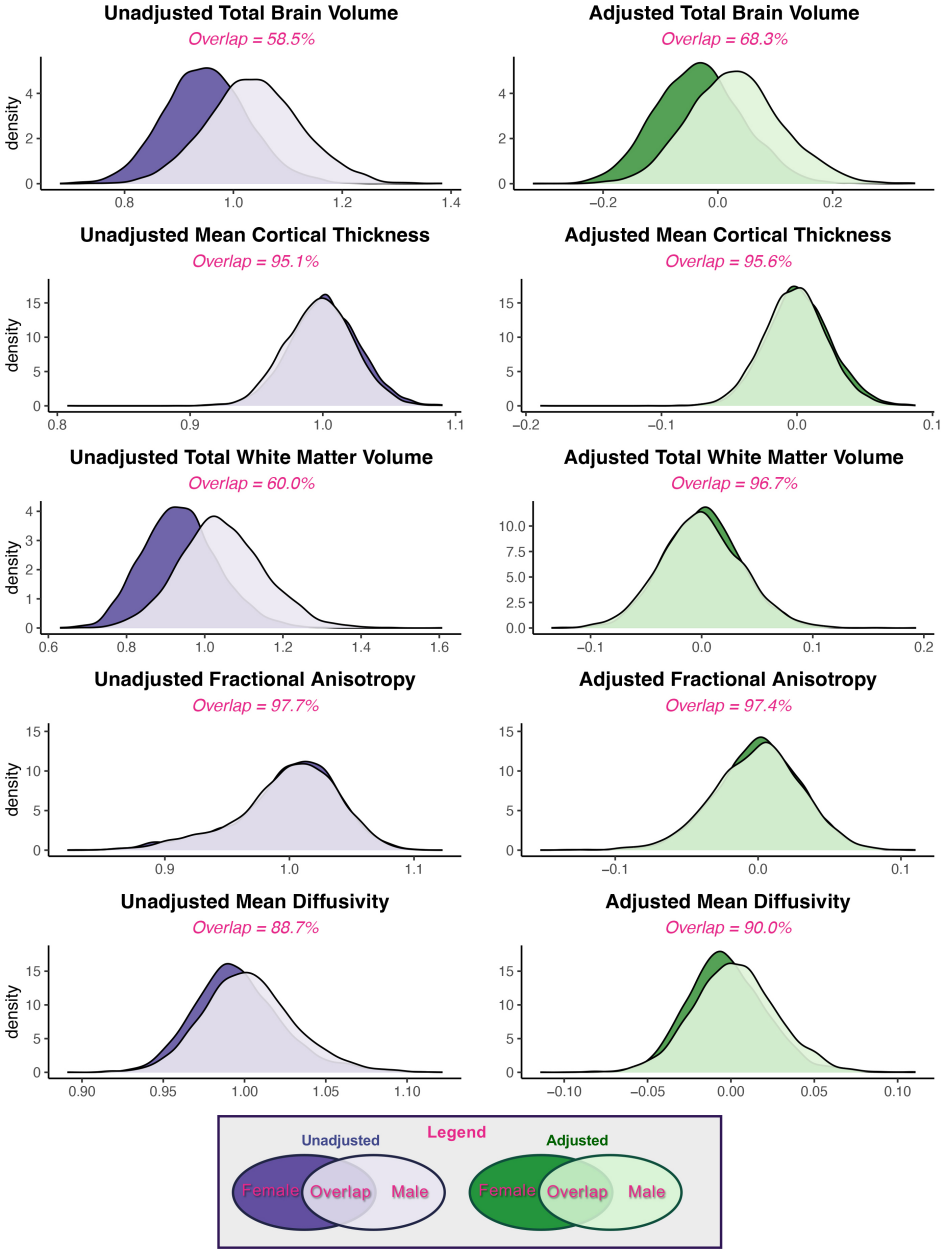
Overlap of global brain metrics in early adolescent males and females. Density plots and overlap of whole-brain measurements for both the unadjusted (purple) and adjusted (i.e., residual estimates, green) estimates for male (light) and female (dark) adolescents. Please note that the x-axis and y-axis change between measures (i.e. between brain volume and FA) due to large differences in scale. Adjusted results were calculated using the residuals of a linear mixed-effects model with age (in months), pubertal development, maximum parental education, race, ethnicity, and scanner manufacturer. Models of white matter volume, FA, and MD also included scaled total brain volume.

In all global measures examined, within-sex variance exceeded the variance between the group means for male and female adolescents. In the unadjusted data, we observed that male variance was greater than female variance for TBV and total white matter volume, while female variance exceeded male variance for mean cortical thickness (Supplementary Table 3). However, the RCV_M_ of unadjusted global measures were similar between males and females for all measures (Supplementary Table 3), suggesting similar dispersion in global brain metrics after accounting for potential scaling differences and outliers. Similarly, for all whole-brain measures—both adjusted and unadjusted—the overlap between the male and female distributions was larger than the portions of the distribution unique to either sex (Fig. 1).

#### 3.1.2 Analytical statistics

Using the Fligner-Killeen test, we found significant inhomogeneity of variance between male and female adolescents regarding TBV and total white matter volume. After adjusting for additional variables, inhomogeneity of variance was significant for TBV, white matter volume, mean FA, and mean MD (Table 3), suggesting that inhomogeneity of variance between the sexes in these metrics may be influenced by other sociodemographic or biological factors not captured in our model. The Cliff’s δ test results found large sex differences in TBV and total white matter volume and small sex differences in MD, but negligible differences in mean cortical thickness and FA. After adjusting for our fixed effects, a medium effect remained for TBV, while differences were negligible for all other measures of global structure (Table 3). Accordingly, the probability that a random male youth would have greater TBV than a random female was 78.1% before adjustment and remained 71.9% after adjustment, while the CLES for other global measures shrunk to less than a 5% difference from chance after adjustment (Table 3).

**Table 3.** Global brain analytical statistics.

Data type	Region of interest	Fligner-Killeen	Fligner-Killeen p-value	Cliff’s δ	Cliff’s δ magnitude	CLES (M > F)
Unadjusted	Total brain volume (mm³)	42.39	**7.49E-11**	0.562	Large	0.781
	Total white matter volume (mm³)	45.73	**1.35E-11**	0.539	Large	0.769
	Whole-brain cortical thickness (mm)	1.01	0.641	0.073	Negligible	0.463
	Fractional anisotropy all tracts	0.20	0.654	-0.008	Negligible	0.496
	Mean diffusivity all tracts	4.99	**0.025**	0.148	Small	0.574
Adjusted	Total brain volume (mm³)	15.51	**8.23E-05**	0.438	Medium	0.719
	Total white matter volume (mm³)	23.00	**1.62E-06**	-0.007	Negligible	0.496
	Whole-brain cortical thickness (mm)	1.94	0.469	0.063	Negligible	0.464
	Fractional anisotropy all tracts	7.04	**0.008**	-0.062	Negligible	0.469
	Mean diffusivity all tracts	12.54	**3.98E-04**	0.052	Negligible	0.526

Results of the Fligner-Killeen test for inhomogeneity of variance and Cliff’s delta (**δ**) test, along with the CLES (the probability that a randomly chosen male adolescent will have an observed value greater than a randomly chosen female adolescent). Adjusted results were calculated using the residuals of a linear mixed-effects model with age (in months), pubertal development, maximum parental education, race, ethnicity, scanner manufacturer and—in the case of total white matter volume, fractional anisotropy, and mean diffusivity—for scaled total brain volume as a fixed effect, and study site as a random effect. Bolded values reflect p-values <0.05, and the magnitude of Cliff’s δ is interpreted based on thresholds provided by Romano et al. (2006).

### 3.2 Regional gray matter and subcortical macrostructure

#### 3.2.1 Descriptive statistics

In all cortical gray matter and subcortical volumes as well as cortical thickness regions examined, the variance between group means was smaller than the within-sex variance using both the unadjusted and adjusted volumes. The RCV_M_ and OVL_2_ for cortical volumes, subcortical volumes, and cortical thickness for male and female adolescents can be found in Supplementary Tables 4-9 and Supplementary Figures 2-4. As expected, adjustment for TBV and other sources of variance led to an increase in the overlap of male and female regional cortical volumes (unadjusted: OVL_2_ range = 0.688–0.921, median = 0.788; adjusted: OVL_2_ range = 0.899–0.972, median = 0.939) and subcortical volumes (unadjusted: OVL_2_ range = 0.659–0.921, median = 0.749; adjusted: OVL_2_ range = 0.896–0.959, median = 0.939). In contrast, adjustment had less of an impact on cortical thickness overlap (unadjusted: OVL_2_ range = 0.865–0.985, median = 0.961; adjusted: OVL_2_ range = 0.900–0.986, median = 0.968).

#### 3.2.2 Analytical statistics

**Fig. 2.**
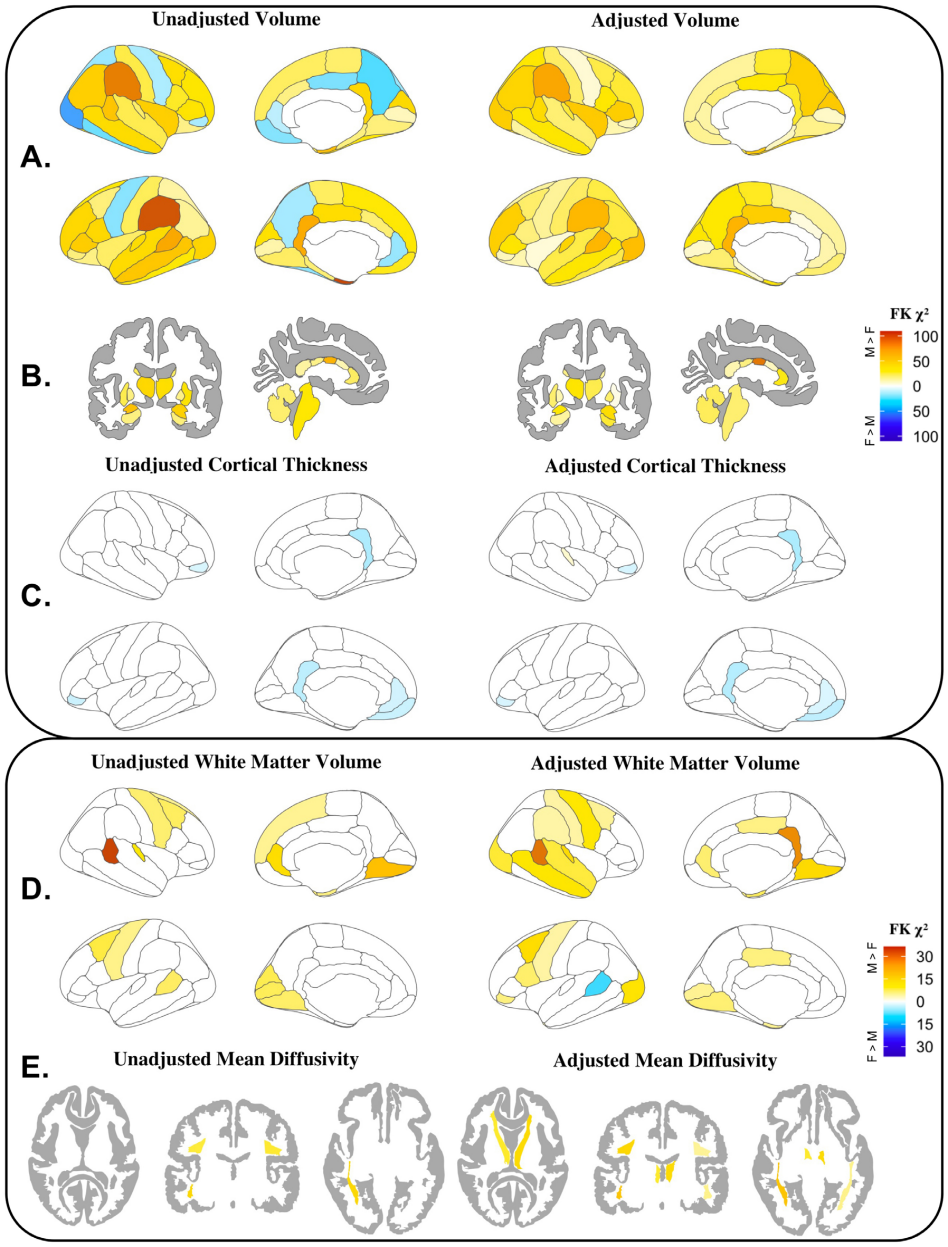
Significant inhomogeneity of variance results between male and female adolescents for unadjusted and adjusted regional measures. (A) Cortical volumes (mm^3^), (B) subcortical volumes (mm^3^), (C) cortical thickness (mm), (D) white matter volumes (mm^3^), and (E) mean diffusivity (mm^2^/s). Colors reflect Fligner-Killeen χ2 statistic: green denotes males > females; red denotes females > males. Values not shown for the volumes of the corpus callosum (anterior, mid anterior, mid posterior, posterior). Adjusted results were calculated using the residuals of a linear mixed-effects model with age (in months), pubertal development, maximum parental education, race, ethnicity, and scanner manufacturer as fixed effects and study site as a random effect. Cortical, subcortical, white matter volume, and MD models also included scaled total brain volume as a fixed effect. Note, results for fractional anisotropy are not presented as inhomogeneity of variance between male and females was only significant for the adjusted left superior longitudinal fasciculus. Yellow/orange = males > females; Blue/purple = females > males.

**Fig. 3.**
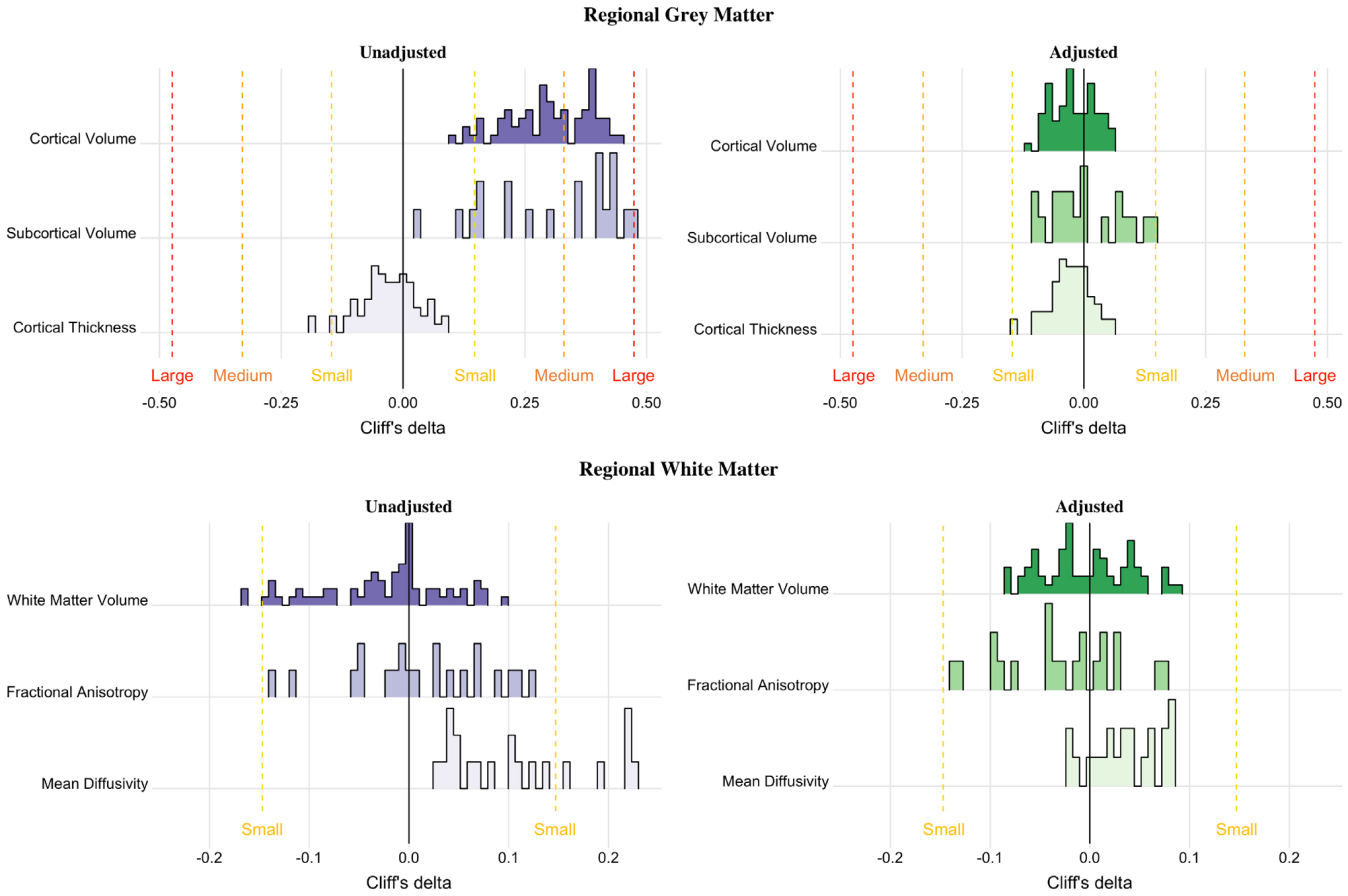
Distribution of Cliff’s delta for unadjusted and adjusted regional measures for gray matter and white matter. The interpretation for the magnitude of Cliff’s delta is indicated by the dashed lines where yellow indicates the threshold for a small effect size, orange indicates medium, and red indicates a large effect size. Adjusted results were calculated using the residuals of a linear mixed-effects model with age (in months), pubertal development, maximum parental education, race, ethnicity, and scanner manufacturer as fixed effects, and study site as a random effect. Models of cortical volumes, subcortical volumes, white matter volumes, FA, and MD also included scaled total brain volume as a fixed effect.

Gray matter showed similar results of inhomogeneity of variance between the sexes using both unadjusted and adjusted regions of interest (Fig. 2). For cortical and subcortical volumes, inhomogeneity of variance between sexes was significant in all regions, with greater variance among male adolescents than among female adolescents (Fig. 2A-B). The greatest sex differences in variance were seen in the volume of the supramarginal gyrus and central corpus callosum. In contrast to volume, female cortical thickness variance significantly exceeded male variance in the left medial orbitofrontal cortex, left rostral anterior cingulate, and bilateral pars orbitalis and isthmus cingulate (Fig. 2C). After adjustment, female cortical thickness variance also significantly exceeded male variance in the right transverse temporal gyrus. Prior to adjustment, Cliff’s δ revealed medium effect sizes for sex differences in cortical volume in 26/68 regions (38%) and an additional 38 regions (56%) met the threshold for a small effect size (range δ = 0.099–0.439, median δ = 0.299) (Fig 3). In the subcortex, the right cerebellum met the threshold for a large effect size (δ = 0.481), 10/20 regions (50%) met the threshold for a medium effect size, and 6/20 regions (30%) met the threshold for a small effect size (range δ = 0.028–0.481, median δ = 0.358) (Fig. 3). In the unadjusted cortical thickness data, Cliff’s δ reached the threshold for a small effect size in the left transverse temporal gyrus (δ = 0.182) and bilaterally in the parahippocampal gyrus (right hemisphere δ = 0.194; left hemisphere δ = 0.147), with all other regional Cliff’s δs below the threshold for small effect size. After adjustment, all sex differences in regional cortical volume, subcortical volume, and cortical thickness were even smaller in magnitude.

**Fig. 4.**
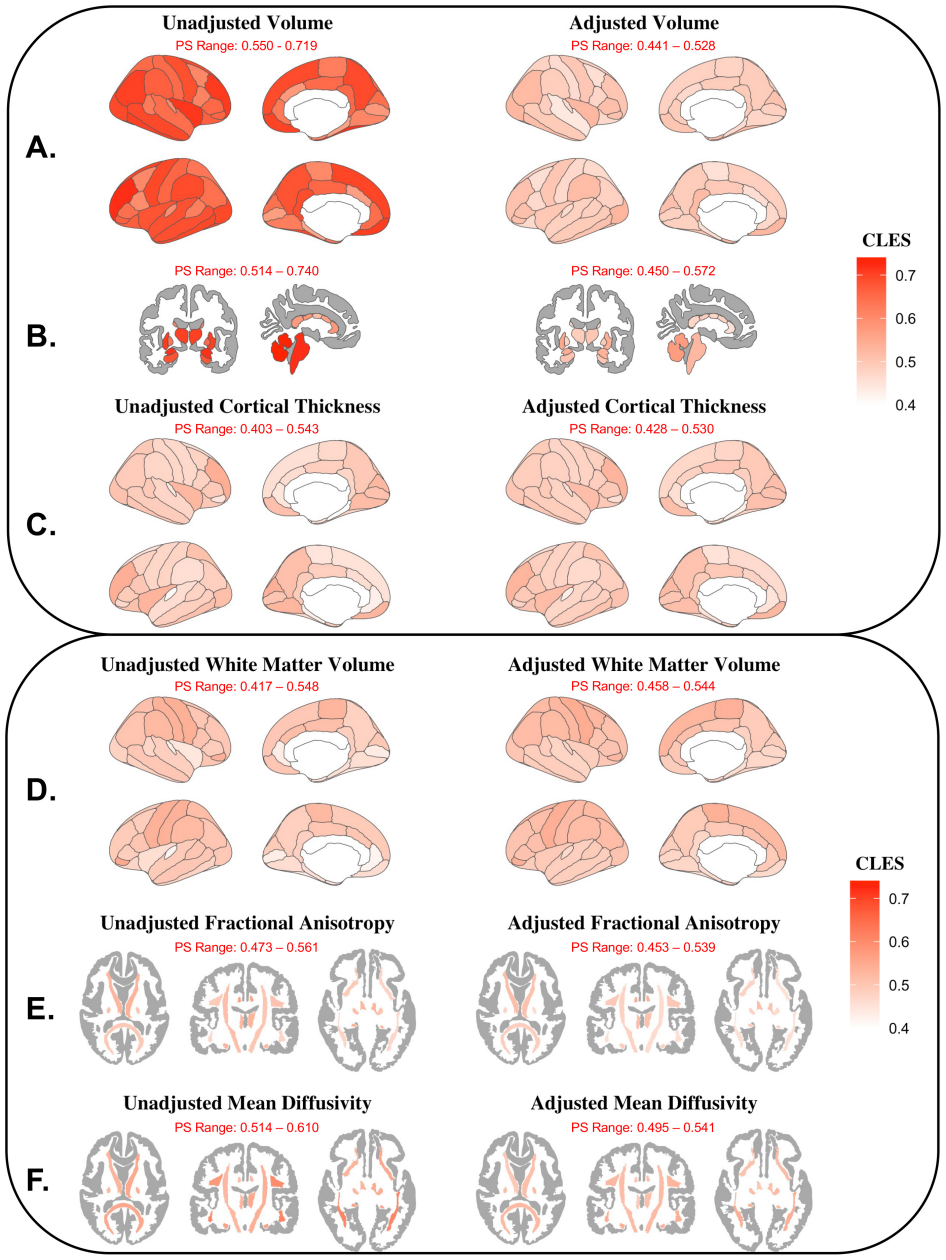
Common language effect size (CLES) for adolescents by regional measure. (A) Cortical volumes (mm^3^), (B) subcortical volumes (mm^3^), (C) cortical thickness (mm), (D) white matter volumes (mm^3^), (E) white matter fractional anisotropy (FA; unitless), and (F) white matter mean diffusivity (MD; mm^2^/s). The CLES describes the probability that a randomly selected male subject will have a value larger than a randomly selected female. Adjusted results were calculated using the residuals of a linear mixed-effects model with age (in months), pubertal development, maximum parental education, race, ethnicity, and scanner manufacturer as fixed effects, and study site as a random effect. Models of cortical volumes, subcortical volumes, white matter volumes, FA, and MD also included scaled total brain volume as a fixed effect.

The CLES in regional cortical volume before adjustment ranged from 0.549 (left parahippocampal gyrus) to 0.719 (left rostral middle frontal gyrus) with a median of 0.649. After adjustment, the probability that a male would have a greater regional volume than a female was closer to chance (range: 0.441–0.528; median = 0.488) (Fig. 4A). In the subcortex, the CLES ranged from 0.514 (central corpus callosum) to 0.740 (right cerebellum) with a median of 0.679. After adjustment, the CLES was reduced (range: 0.450–0.572; median = 0.495) (Fig. 4B). Regarding regional cortical thickness, the CLES findings suggested that in most unadjusted regions (49/68) males had a lower than 50% probability of having greater cortical thickness than females. The CLES prior to adjustment ranged from 0.403 (right parahippocampal gyrus) to 0.542 (right rostral middle frontal gyrus) with a median of 0.484. After adjustment, the CLES values were similar (range: 0.428–0.530; median = 0.486) (Fig. 4C).

### 3.3 Regional white matter volume

#### 3.3.1 Descriptive statistics

In all regions examined, the variance in white matter volumes between sexes was smaller than the within-sex variance (Supplementary Tables 10-11). RCV_M_ for each sex for all white matter regions can be found in Supplementary Figure 5. Overlap coefficients were similar before and after adjustment (unadjusted: OVL_2_ range = 0.879–0.987, median = 0.963; adjusted: OVL_2_ range = 0.928–0.984, median = 0.963).

#### 3.3.2 Analytical statistics

Adjusting for additional covariates increased the percentage of regions with significant inhomogeneity of variance (unadjusted: p *<* 0.05 in 23.5% of regions; adjusted: p *<* 0.05 in 41.2% of regions) (Fig. 2D). Where significant, males showed greater regional variance than females, except in the banks of the left superior temporal sulcus, where female variance exceeded male variance. In the unadjusted data, Cliff’s δ reached the threshold for a small effect size in the left transverse temporal gyrus (δ = 0.166) and the left rostral anterior cingulate (δ = 0.162), with all other regional effect sizes were negligible (i.e., δ < 0.15). After adjustment, all regional white matter volume sex differences were negligible (Fig. 3). CLES results showed that in most unadjusted regions (46/68), the probability of males having larger white matter volumes than females was below 50%. Prior to adjustment, the CLES ranged from 0.417 (left transverse temporal gyrus) to 0.548 (left pars orbitalis) with a median of 0.493 (Fig. 4D). After adjustment, the CLES values were closer to chance (range: 0.458–0.544; median = 0.496) (Fig. 4D).

### 3.4 White matter tract microstructure

#### 3.4.1 Descriptive statistics

In both regional FA and MD, the variance between the male and female mean values was universally smaller than the within-sex variance (Supplementary Tables 12-15). RCV_M_ for FA and MD for both sexes for each white matter tract is shown in Supplementary Figures 6-7. High overlap was observed in both the raw and adjusted FA (unadjusted: OVL_2_ range = 0.893–0.981, median = 0.961; adjusted: OVL_2_ range = 0.899–0.984, median = 0.966) as well as MD (unadjusted: OVL_2_ range = 0.829–0.967, median = 0.920; adjusted: OVL_2_ range = 0.928–0.977, median = 0.956) (Supplementary Tables 12-15).

#### 3.4.2 Analytical statistics

Interestingly, adjustment increased the number of regions with significant inhomogeneity of variance in FA and MD. No white matter tracts showed significant inhomogeneity of variance in FA before adjustment, but the left superior longitudinal fasciculus showed significant inhomogeneity after adjustment (Supplementary Table 13). Before adjustment, male youth displayed significantly greater variance in MD than female youth in the left inferior longitudinal fasciculus, left inferior fronto-occipital fasciculus, and bilaterally in the superior longitudinal fasciculus (Fig. 2E). After adjustment, MD variance differed significantly between sexes in the corpus callosum, right uncinate fasciculus, and bilaterally in the superior longitudinal fasciculus, fornix, anterior thalamic radiations, inferior longitudinal fasciculus, and the inferior fronto-occipital fasciculus (12/21 ROIs; Fig. 2E).

Cliff’s δ values for regional FA were all below 0.147 both before and after adjustment, indicating negligible sex differences (Fig. 3). Prior to adjustment, Cliff’s δ revealed small sex differences in MD bilaterally in the inferior longitudinal fasciculus (right hemisphere δ = -0.219; left hemisphere δ = -0.219) and inferior fronto-occipital fasciculus (right hemisphere δ = -0.229; left hemisphere δ = -0.220); however, after adjustment, all sex differences in MD were negligible (i.e., δ < 0.147). The CLES for FA prior to adjustment ranged from 0.432 (right inferior fronto-occipital fasciculus) to 0.561 (left cingulate cingulum) with a median of 0.504 (Fig. 4E). After adjustment, the probabilities remained relatively close to chance (range: 0.433–0.539; median = 0.487) (Fig. 4E). The CLES for MD prior to adjustment ranged from 0.514 (forceps major) to 0.615 (left inferior fronto-occipital fasciculus) with a median of 0.550 (Fig. 4F). After adjustment, the probabilities were closer to chance (range: 0.489–0.542; median = 0.520) (Fig. 4F).

#### 3.4.3 Sensitivity analysis

Our volumetric and white matter microstructure models all included TBV as a fixed effect. However, we also conducted an additional set of white matter sensitivity analyses adjusting for all fixed and random effects, excluding TBV. The results were nearly identical to those adjusting for TBV (Supplementary Tables 13 & 15), including the increase in the number of brain regions showing significant inhomogeneity of variance for FA and MD after adjusting for covariates.

Our second sensitivity analysis adjusted for all the original fixed and random effects, excluding pubertal development. We found that removing pubertal development from the mixed-effects model resulted in much more residual variance for both males and females in cortical gray matter volumes, but had only minor impacts on residual male and female variance for subcortical volume, white matter volume, cortical thickness, FA, or MD. The impact on the probability of superiority results was similarly small. In most regions, removing pubertal development from the model did not impact the interpretation of the Fligner-Killeen test or Cliff’s delta test results. Nonetheless, in the left transverse temporal and bilateral parahippocampal gyrus, the size of the sex difference in cortical thickness increased from negligible to small. Similarly, the sex difference in cerebellar volume and bilateral inferior fronto-occipital fasciculus FA increased from negligible to small. The sensitivity analysis also found that overlooking pubertal development caused sex differences in variance to fall below the threshold of significance (p < 0.05) for the right inferior temporal and right fusiform gyrus volume, left rostral anterior cingulate cortical thickness, and right postcentral white matter volume. In contrast, the sex difference in variance became significant for corpus callosum FA and MD when pubertal development was ignored.

## 4 Discussion

This study contextualizes previous reports of widespread group mean sex differences previously reported in early adolescence (Jamieson et al., 2023; Kurth et al., 2021; Lawrence et al., 2023; Lenroot et al., 2007; Peper et al., 2009; Raznahan et al., 2011; Torgerson et al., 2024) by comparing the within- and between-sex variance and quantifying the neuroanatomical similarities between the sexes at ages 9 to 11 years old. We observed high overlap between male and female distributions on all measures and found greater dispersion among male youth in most measures of cortical, subcortical, and white matter volume. In line with previous research in the developing brain (Bottenhorn et al., 2023; Forde et al., 2020; Wierenga et al., 2018), we detected significant inhomogeneity of variance between male and female youths in regional cortical and subcortical volume. In addition, the number of brain regions showing significant inhomogeneity of variance increased for white matter outcomes after adjusting for other covariates, suggesting potential sex differences in potential covariate–outcome relationships when it comes to brain outcomes (Buckley et al., 2017). Moreover, using Cliff’s delta, we found the effect size for sex differences to be negligible in all regional measures of brain structure after adjusting for other sources of variance, such as TBV. We conclude that mean group sex differences in early adolescent brain structure are considerably smaller than the sex similarities indicated by the overlap coefficient and therefore do not reflect distinct sex-based phenotypes (e.g., sexual dimorphism). Holistically, these results underscore the importance of testing model assumptions—including inhomogeneity of variance between sexes—and choosing appropriate non-parametric statistical methods when probing sex differences in brain morphology.

To quantify similarity, we calculated the overlap (OVL_2_) between male and female distributions and the common language effect size (CLES) in each global and regional measure. Male and female total brain volume (TBV) distributions showed the least overlap between sex distributions of any measure examined, both before and after adjustment (raw OVL_2_ = 0.585; corrected OVL_2_ = 0.682). Furthermore, TBV was the only measure whose Cliff’s delta effect size was non-negligible after adjustment for other sources of variance (δ = 0.438; medium effect size). This further supports its status as the largest and most replicable sex difference in pediatric brain structure (Ducharme et al., 2016; Lenroot et al., 2007; Levenstein et al., 2023; Paus et al., 2010; Sussman et al., 2016). However, brain size is related to overall body composition (Burger et al., 2018; Schoenemann, 2004), so this difference may be at least partly a reflection of overall body composition differences between male and female adolescents. Across all adjusted regional structural metrics examined, a minimum of 89.6% of all youths fell within the overlapping portion of the joint distribution. Regional CLES values for males were close to chance (range: 0.428–0.572). Similar results have been shown in adults, where “extensive overlap” has been reported between male and female distributions in all brain regions examined (Joel et al., 2015). These findings further demonstrate that the brains of male and female youth appear very similar after accounting for additional sources of variance in the data. Therefore, our results extend the conclusions of Joel et al. (2015) to early adolescents and reaffirm that human brain macrostructure does not exist in binary, sexually dimorphic categories associated with sex, nor does it appear to exist on a continuum between male and female extremes.

This work expands upon previous findings of sex differences in within-sex variability in childhood (Bottenhorn et al., 2023; Wierenga et al., 2018). Wierenga et al. reported greater male variability in gray matter volume, whereas Bottenhorn et al. found greater male variability in white matter change over time, but greater female variability in cortical macro- and micro-structural change over time. After adjustment, we found significant sex differences in variance for TBV, average FA, average MD, and all regional cortical and subcortical volumes, with large inhomogeneity in the parietal lobe, basal ganglia, and limbic regions. Male variance exceeded female variance in all cortical and subcortical volume regions both before and after adjustment. Higher male variability in volume may be due, in part, to random X chromosome inactivation: heterozygous females express two different alleles of a single gene in a mosaic pattern throughout the brain, whereas homozygous females and males with a single X chromosome exhibit uniform expression (Raznahan et al., 2018; Raznahan & Disteche, 2021). Consequently, if two alleles of an X-chromosome gene have opposite effects, males and homozygous females will exhibit one of two extreme phenotypes, while heterozygous females will exhibit a mixed phenotype, decreasing the average trait variability among females. These results suggest that male structural variability is greater than female structural variability in gray matter volume and white matter microstructure, whereas female variability exceeds male variability in cortical thickness. Therefore, future research should examine the link between X-chromosome genes and regional gray matter volumes, while other sources of sex-related variance—such as estrogen and testosterone differences (Herting et al., 2015; Savic et al., 2017), BMI (Laurent et al., 2020), aerobic fitness (Chaddock-Heyman et al., 2015; Ruotsalainen et al., 2020), or eating behaviors (Breton et al., 2024) —should be explored with regard to cortical thickness variance.

Many univariate methods of comparison (i.e., t-tests, ANOVA) rely on assumptions of normality and homogeneity of variance. Consequently, such tests are inappropriate for comparing sexes on measures with significant inhomogeneity of variance between sexes, such as cortical and subcortical volume. Thus, we used a robust, non-parametric method that does not assume equal variance (Cliff’s delta) to assess the magnitude of sex differences across the entire distribution. In the unadjusted data, we found widespread regional sex differences in cortical and subcortical volume with small and medium effect sizes and a large difference in the right cerebellum. A few unadjusted cortical thickness, white matter volume, MD regions also showed small effect sizes, but all FA effects were negligible. When looking at the data adjusted for other sources of variance, all regional measures of sex differences were negligible in magnitude. Sex differences in TBV retained a medium effect size after adjustment. Still, in 28.1% of cases, a randomly chosen female adolescent would have a larger brain than a randomly chosen male adolescent (CLES = 0.719). The ubiquity of the high overlap, low Cliff’s delta values, and low CLES after adjustment could suggest that most sex differences are better attributed to other variables – such as TBV, age, or puberty. Our sensitivity analyses further reaffirmed the importance of accounting for known sources of variance—particularly TBV or intracranial volume—when assessing sex differences in brain structure (Eliot et al., 2021; Pintzka et al., 2015; Sanchis-Segura et al., 2020). Failure to account for pubertal development or TBV led to more unexplained variance in the data and inflated the significance of sex differences in several regionional measures.

Taken together, these results contradict claims of sexual dimorphism in pediatric brain structure and contextualize the discussion of sex differences. This distinction between sexual dimorphism and sex differences is meaningful not just in theory, but also in practice. The putative sexual dimorphism of the developing brain has been cited in arguments for single-sex education (Bigler & Signorella, 2011; Eliot, 2013; Halpern et al., 2011) and as evidence in court cases regarding the rights of juveniles (Kennedy, 2021; Re Alex: Hormonal Treatment for Gender Identity Dysphoria, 2004). Yet, the combination of low Cliff’s delta and significant inhomogeneity of variance reported here indicate that average pediatric sex differences in regional brain volume are likely due to disparities in variability rather than two distinct phenotypes with a large mean difference. When there are extensive differences within groups and relatively small differences between groups, membership in the group provides very little information about the trait in question (Anastasi, 1981; Sanchis-Segura & Wilcox, 2024). This lends credence to arguments that conventional methods for preclinical and clinical research of sex differences are not well-designed for application to personalized medicine and are insufficient to address health disparities between males and females (DiMarco et al., 2022; Miller et al., 2015; Richardson et al., 2015). Therefore, our work provides a benchmark grounded in within-sex variability to help contextualize the magnitude of between-sex variability. Future research designs should employ more robust statistical methods and focus on precise sex-linked variables, such as hormones, chromosomes, gene expression, body size and composition, or social determinants of health.

### 4.1 Limitations

Due to the cross-sectional nature of this study and the narrow age range of the participants, our results are limited in scope. As such, they should not be assumed to generalize to brain structure in early childhood, later in adolescence, adulthood or to longitudinal trajectories of brain development. Instead, they offer an in-depth look at the neuroanatomy of children between 9 and 11 years old. We expect sex differences to increase throughout pubertal development, particularly differences in white matter volume, which continues to grow during adolescence and is related to testosterone levels (Bethlehem et al., 2022; Herting et al., 2014; Lian et al., 2025). However, given the large amount of inter-individual variability in pubertal development, it is possible that puberty increases within-sex variance as much or more than it increases between-sex variance.

Furthermore, although sex is multifaceted and encompasses multiple hormonal, genetic, and gross anatomical features, we chose to focus on the presence or absence of a Y chromosome for our operational definition of sex. Consequently, it is unclear to what extent factors like hormone levels, gene expression, or X-chromosome inactivation play a role in our results. Additionally, as a non-experimental study, we cannot provide evidence of a causal link between sex chromosomes and variance. Since few studies examine the influence of social and environmental factors on neuroanatomical sex differences, some authors instead use the term “sex/gender” (Eliot et al., 2021; van Anders, 2022). While our previous work with data from the ABCD Study showed felt-gender did not explain a significant amount of variance in gray or white matter structure (Torgerson et al., 2024), we cannot rule out the possible influence of other sociocultural factors that may be correlated with sex.

Although this study discusses significance for inhomogeneity of variance in terms of p-values (corrected for multiple comparisons), statisticians increasingly warn against dichotomous interpretations of results (i.e., “significant” or “nonsignificant”) (Gagnier & Morgenstern, 2017; Hoekstra et al., 2006) and overreliance on statistical significance to infer practical significance (Bangdiwala, 2016; Mohajeri et al., 2020). Particularly in large, diverse samples, p-values alone may not be reliable indicators of practical significance (Dick et al., 2021; Warwick, 2001). This underscores the danger of dichotomous interpretation of statistical tests in large samples. As such, the significance of the inhomogeneity of variance results should also be interpreted with caution and with the relative magnitude of the statistic itself in mind. Similarly, the interpretation of effect sizes is highly dependent upon context—including the specific outcomes under study, their implications for health or behavior, and the societal or clinical impact of observed differences. Small or negligible effects may still hold significant relevance in certain contexts, such as cumulative or subtle long-term impacts, and small effect sizes are to be expected in brain research in particular (Dick et al., 2021; Owens et al., 2021; Saragosa-Harris et al., 2022).

Moreover, the results may not be directly comparable between brain regions or metrics with very different mean outcomes (i.e., cerebellum volume vs. pars orbitalis volume, average cortical thickness vs. average FA). While this issue is frequently circumvented with standardization, we did not use this technique because it would have altered the variance we sought to characterize. Scaling was similarly rejected because of the associated reduction in significant digits for some measures. For example, when large values (such as TBV in mm^3^) are reduced to a smaller value (such as TBV in m^3^), the loss of precision could lead to more ties when computing the dominance matrix for Cliff’s delta. Therefore, because of the regional differences in scale and the intrinsic link between the mean and variance, caution is urged when comparing results between different brain region outcomes.

### 4.2 Conclusions

Early adolescent male and female brains are more similar than they are different. Due to high within-sex variability, the distributions of males and females have more overlap than difference on all measures of global and regional gray and white matter structure examined. Although male and female adolescents exhibited significant inhomogeneity of neuroanatomical variance, Cliff’s delta demonstrated that sex had a negligible effect on average brain structure. Overall, these results illustrate that sex differences in early adolescent brain structure do not amount to qualitative differences (e.g., sexual dimorphism), and that quantitative differences between sexes are likely too small to be practically meaningful compared with individual variability.

## Supplementary Material

Supplementary Figures

Supplementary Tables

## Data Availability

Data from the ABCD Study Data Release are available for access through the NIMH Data Archive (NDA 3.0 data release 2020: https://doi.org/10.15154/1520591; NDA 4.0 data release 2021; https://doi.org/10.15154/1523041). Only researchers with an approved NDA Data Use Certification (DUC) can obtain ABCD Study data.
