# Supplementary material for: More similarity than difference: Comparison of within- and between-sex variance in early adolescent brain structure
Source: Imaging Neurosci (Camb). 2025 Sep 2;3:IMAG.a.127. doi: 10.1162/IMAG.a.127 (PMC12406053; doi:10.1162/IMAG.a.127)

Supplemental Figures

Supplemental Figure 1.

A graphical representation of exclusionary criteria and the quality control process for the study.

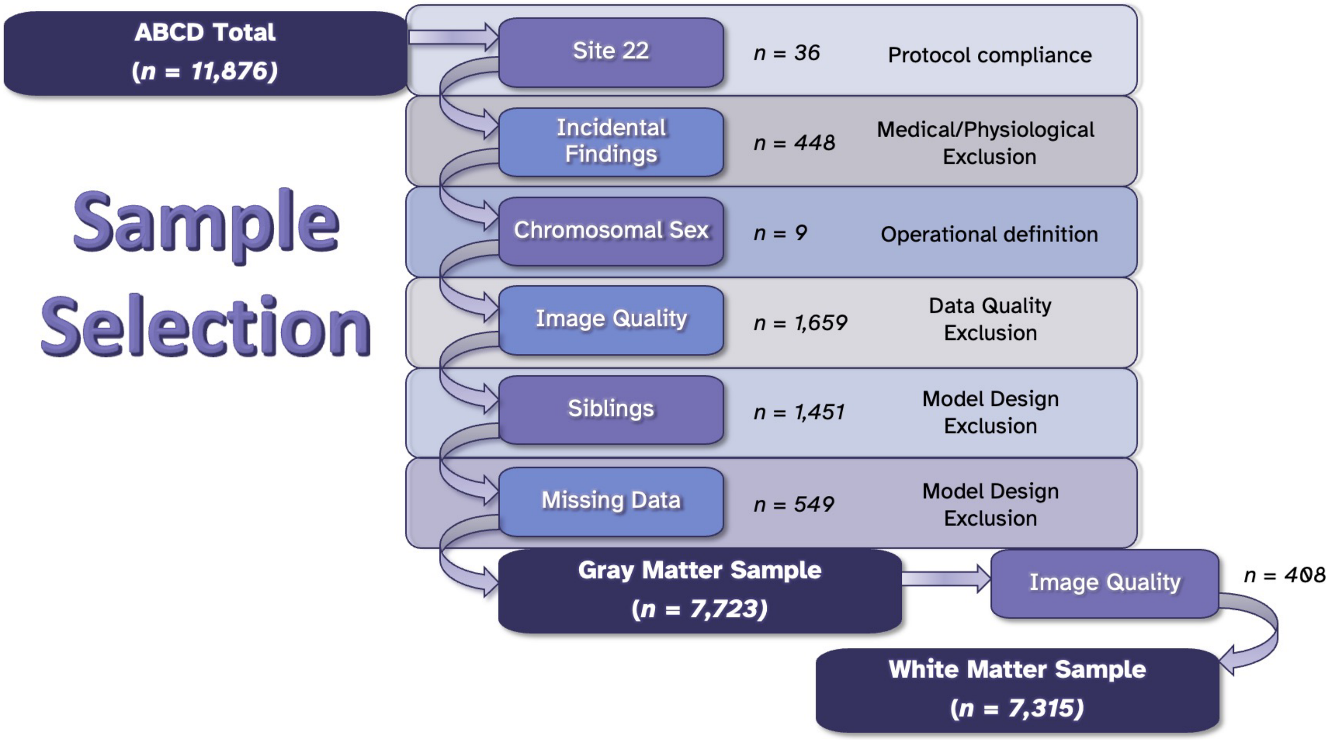

Supplemental Figure 2. ***Unadjusted robust coefficient of variation for males (blue) and females (pink) in regional cortical gray matter volume ROIs.*** *Abbreviations: L = left hemisphere, R = right hemisphere. * denotes Fligner-Killeen χ2 test for inhomogeneity of va*riance FDR p-value < 0.05.

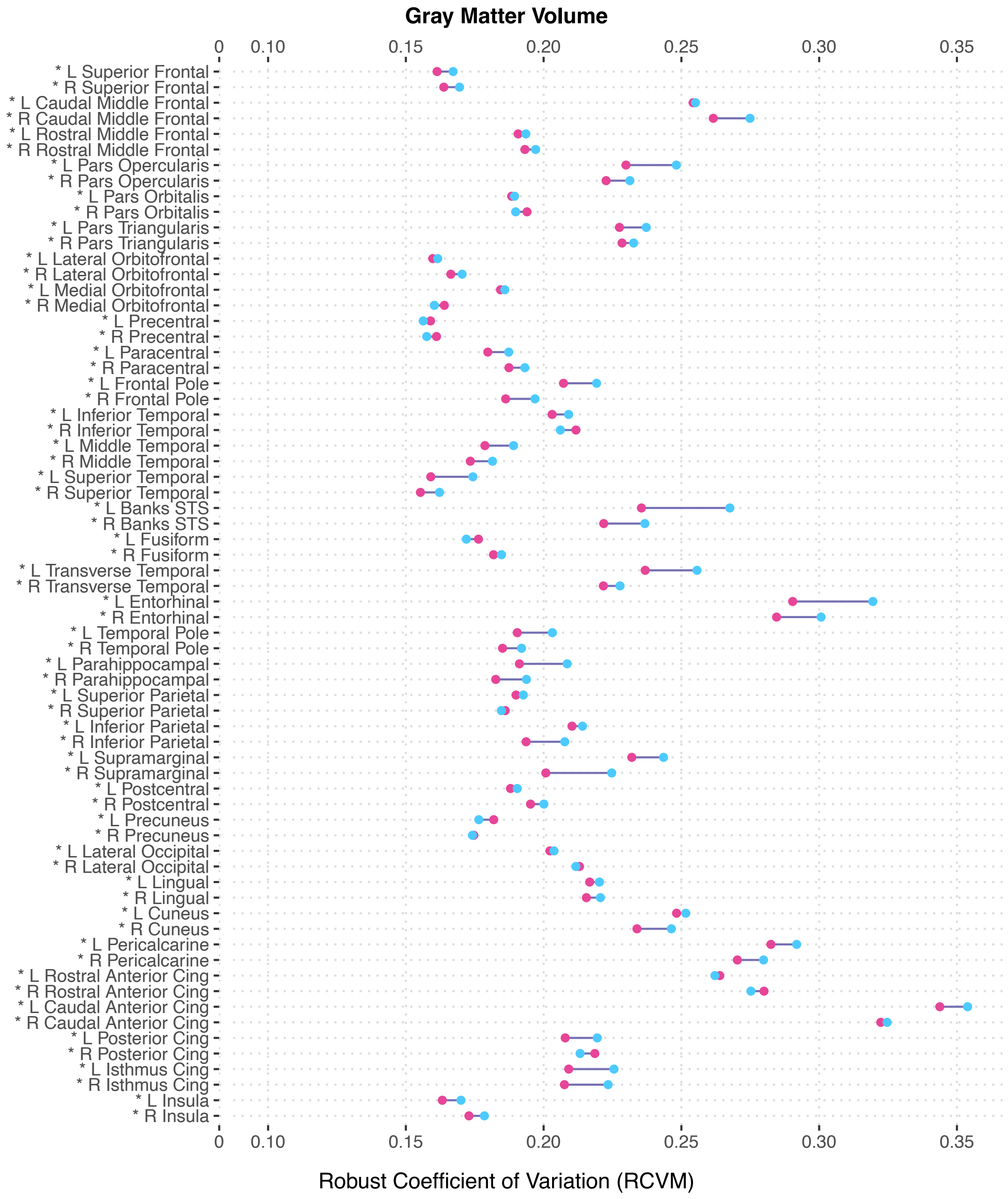

Supplemental Figure 3. ***Unadjusted robust coefficient of variation for males (blue) and females (pink) in regional volumes for subcortical ROIs .*** *Abbreviations: CC = corpus callosum; L = left hemisphere, R = right hemisphere. * denotes= Fligner-Killeen χ2 test for inhomogeneity of variance FDR p-value < 0.05.*

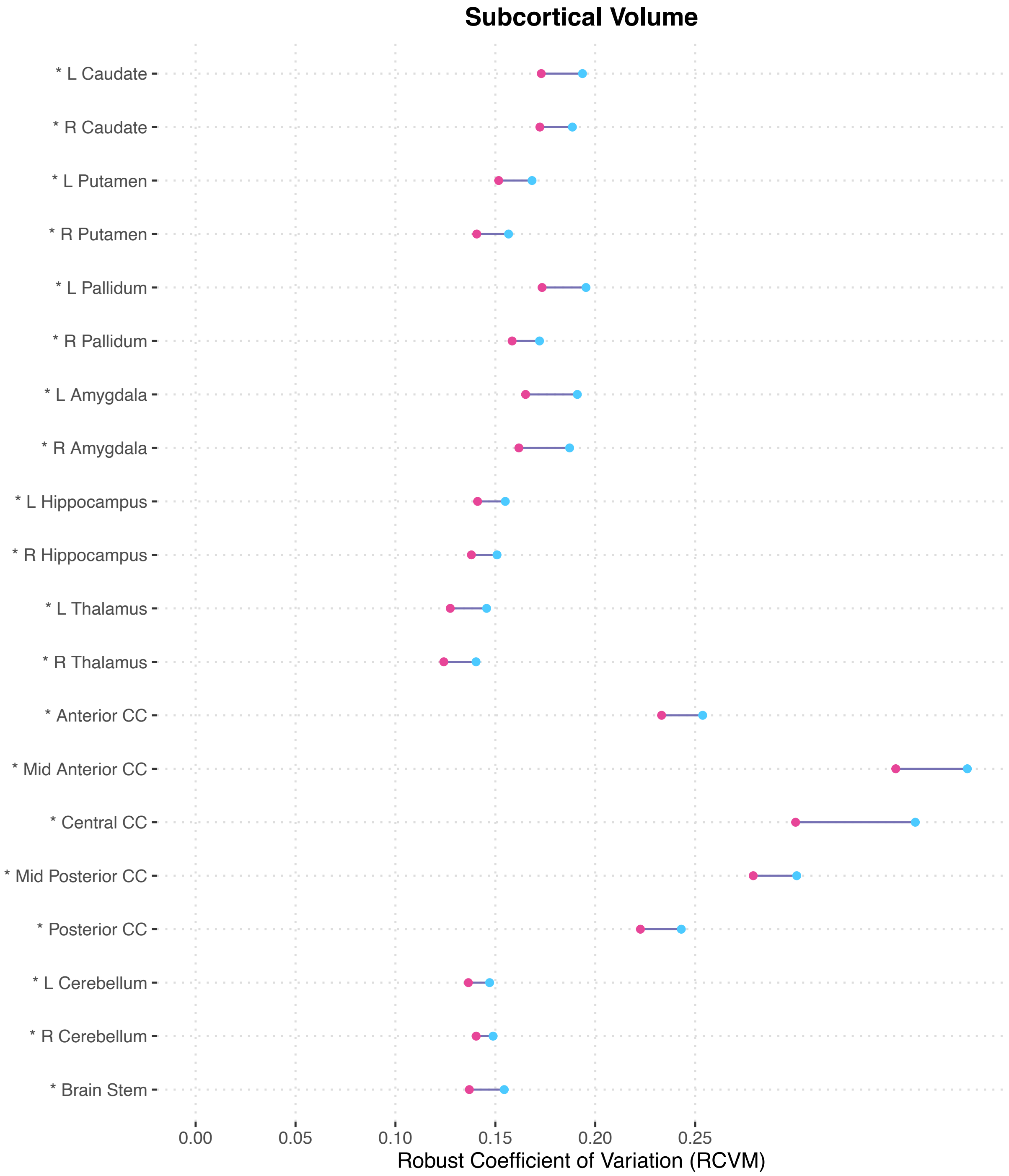

Supplemental Figure 4. ***Unadjusted robust coefficient of variation for males (blue) and females (pink) in regional cortical thickness ROIs.*** *Abbreviations: L = left hemisphere, R = right hemisphere. * denotes= Fligner-Killeen χ2 test for inhomogeneity of variance FDR p-value < 0.05.*

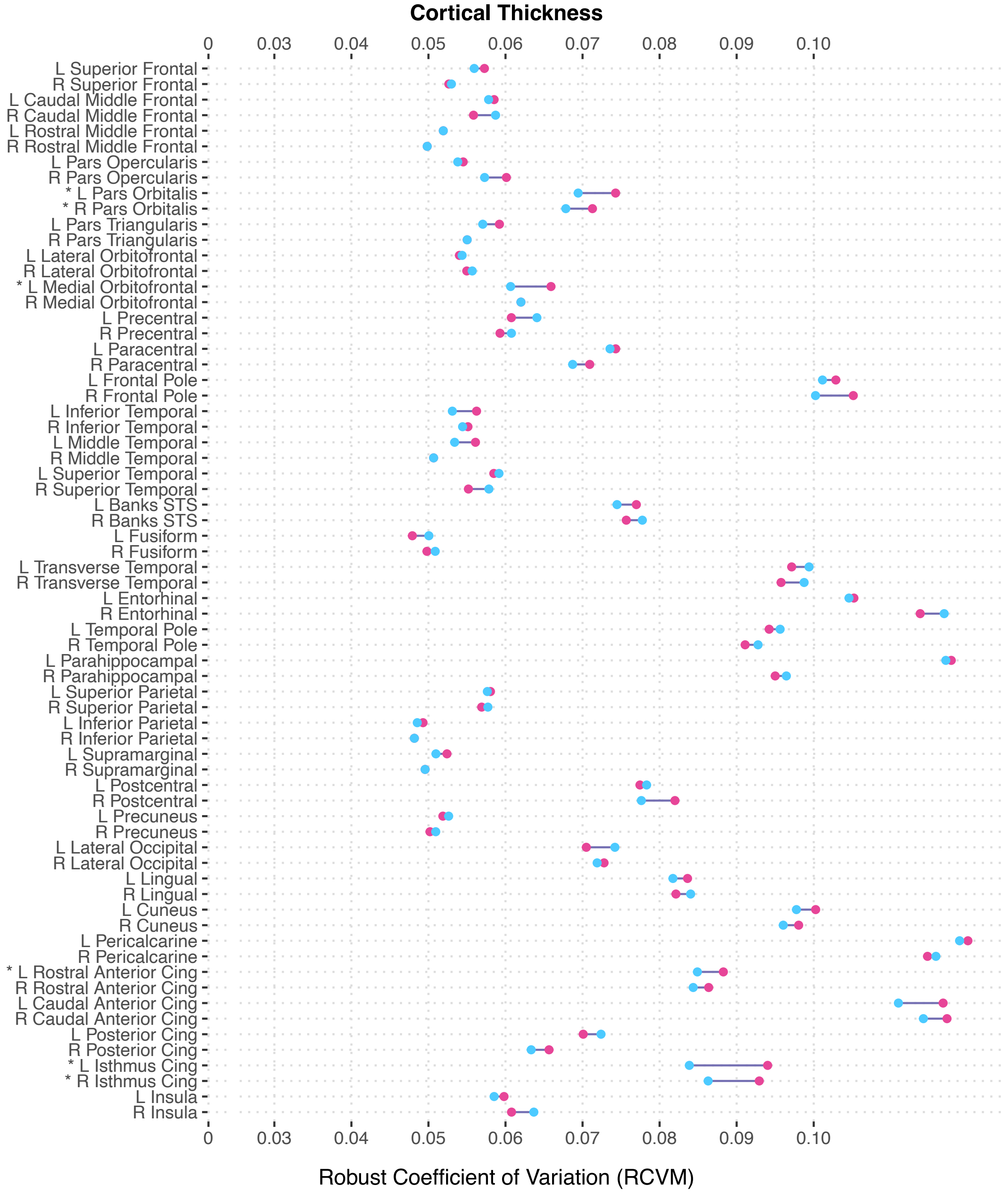

Supplemental Figure 5. ***Unadjusted robust coefficient of variation for males (blue) and females (pink) in regional white matter volume ROIs.*** *Abbreviations: L = left hemisphere, R = right hemisphere.* ***** denotes Fligner-Killeen χ^2 test for inhomogeneity of variance FDR p-value < 0.05.

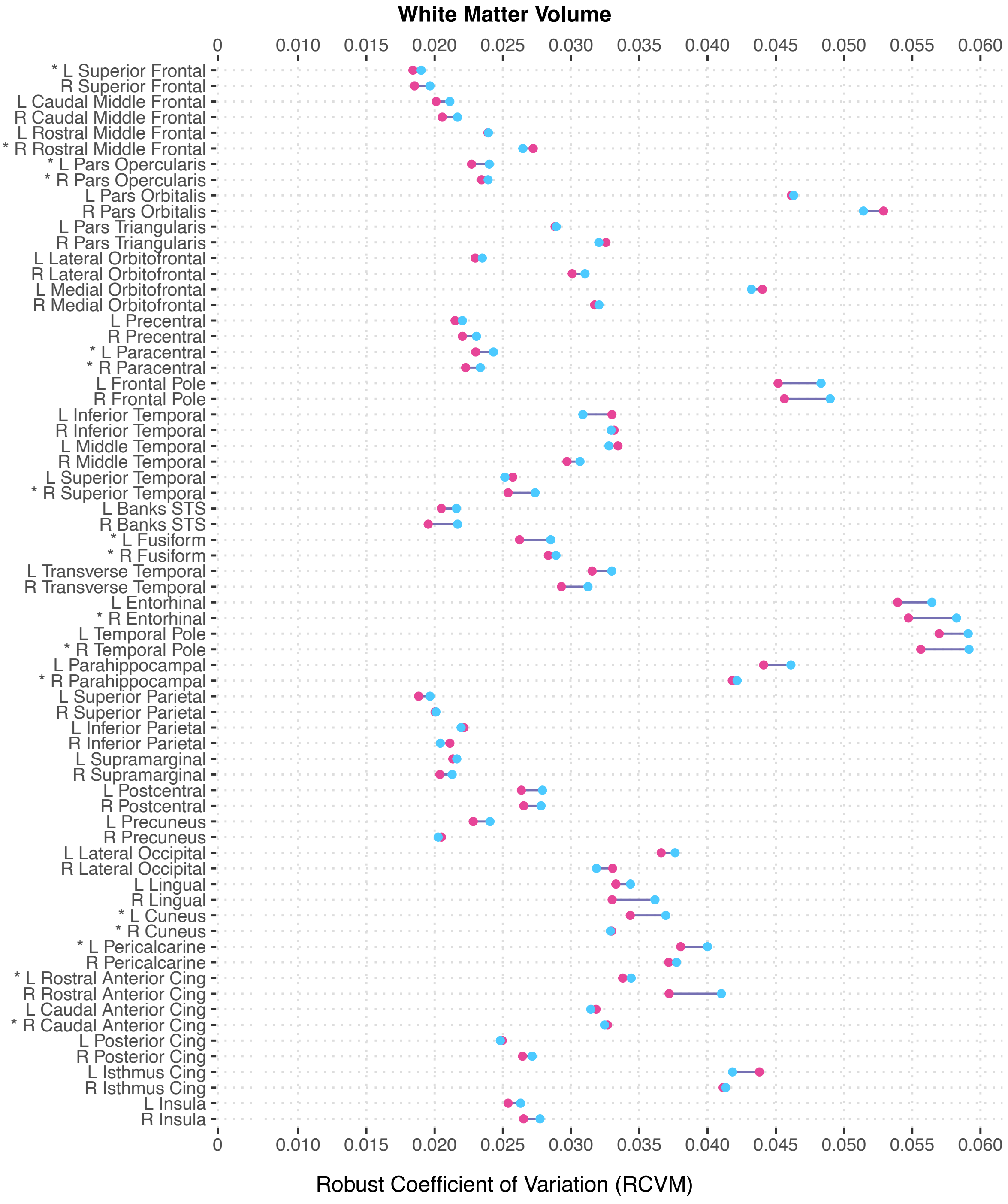

Supplemental Figure 6. ***Unadjusted robust coefficient of variation for males (blue) and females (pink) in regional FA.*** *Abbreviations: L = left hemisphere, R = right hemisphere, CST = corticospinal tract, ILF = inferior longitudinal fasciculus, IFOF = inferior fronto-occipital fasciculus, SLF = superior longitudinal fasciculus.*

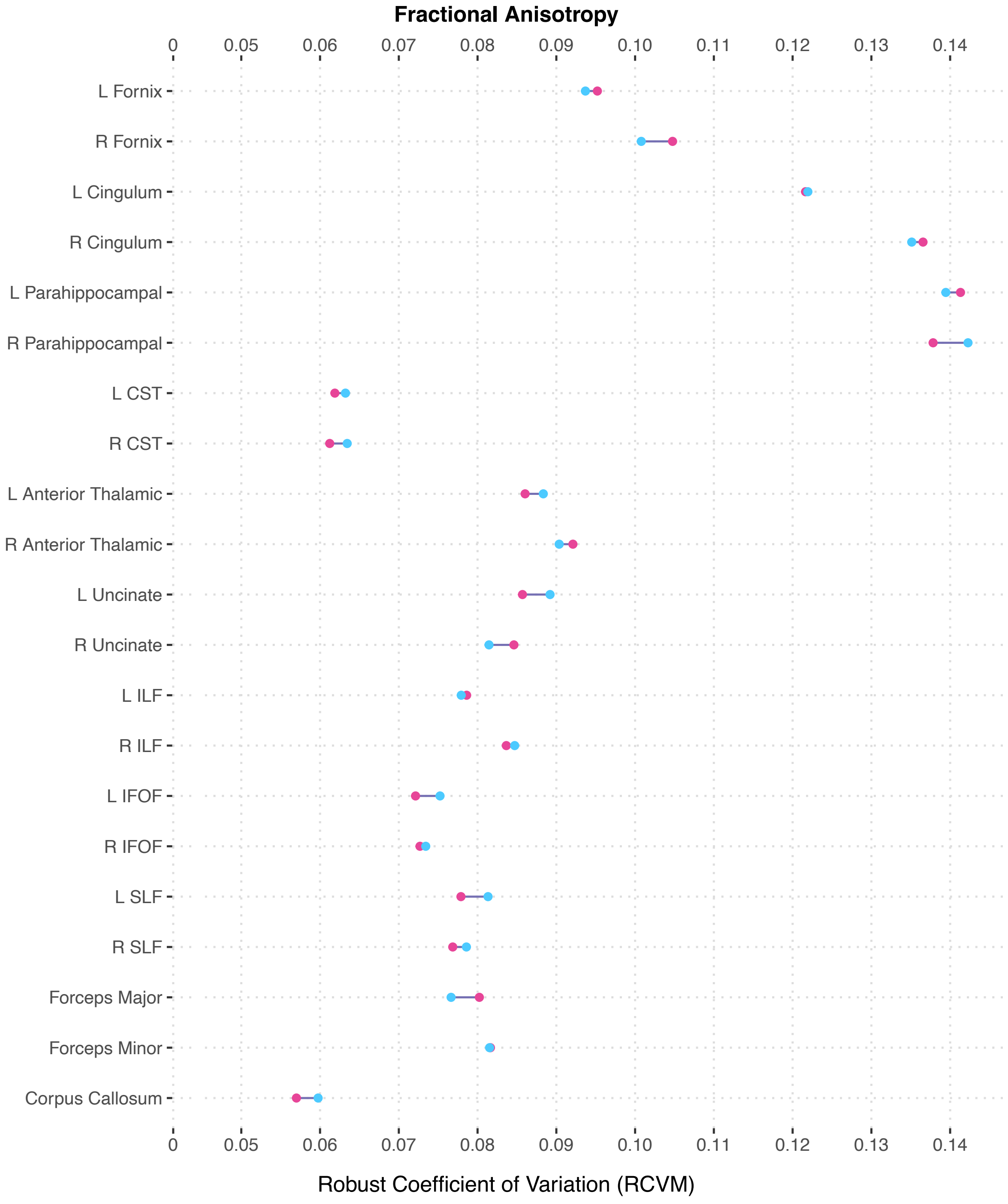

Supplemental Figure 7. ***Unadjusted robust coefficient of variation for males (blue) and females (pink) in regional MD.*** *Abbreviations: L = left hemisphere, R = right hemisphere, CST = corticospinal tract, ILF = inferior longitudinal fasciculus, IFOF = inferior fronto-occipital fasciculus, SLF = superior longitudinal fasciculus.* ***** denotes Fligner-Killeen χ^2 test for inhomogeneity of variance FDR p-value < 0.05.

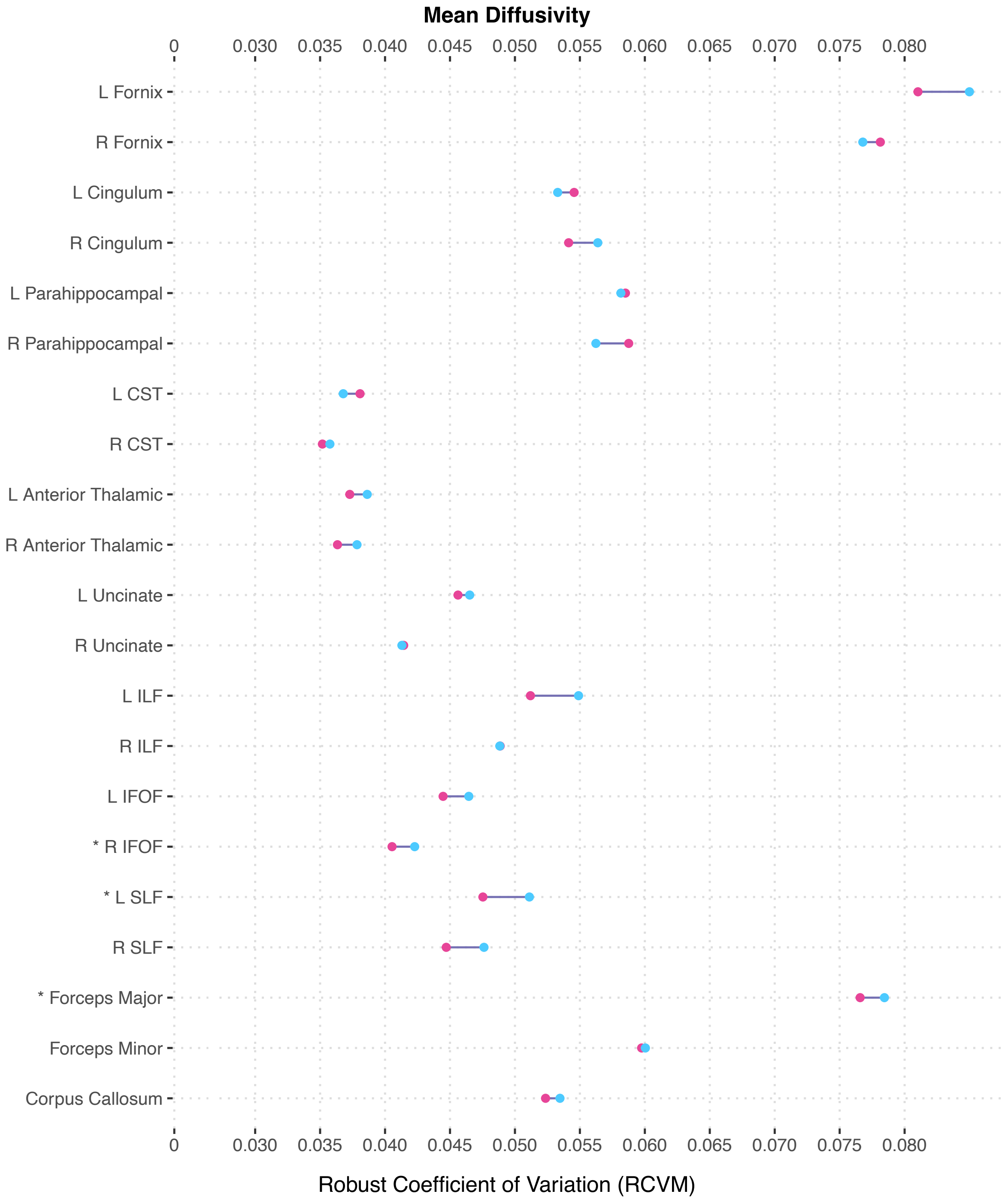

Supplement: Supplementary Figures [file IMAG.a.127_supp.pdf]
